# Effects of Physical Tracing on Estimates of Loss to Follow-Up, Mortality and Retention in Low and Middle Income Country Antiretroviral Therapy Programs: A Systematic Review

**DOI:** 10.1371/journal.pone.0056047

**Published:** 2013-02-12

**Authors:** James H. McMahon, Julian H. Elliott, Steven Y. Hong, Silvia Bertagnolio, Michael R. Jordan

**Affiliations:** 1 Infectious Diseases Unit, Alfred Hospital, Melbourne, Victoria, Australia; 2 Department of Public Health and Community Medicine, Tufts University School of Medicine, Boston, Massachusetts, United States of America; 3 Department of Medicine, Monash University, Melbourne, Victoria, Australia; 4 Burnet Institute, Melbourne, Victoria, Australia; 5 Division of Geographic Medicine and Infectious Diseases, Tufts Medical Center, Boston, Massachusetts, United States of America; 6 University College London, London, United Kingdom; National Institute of Allergy and Infectious Diseases, United States of America

## Abstract

**Background:**

A large proportion of patients receiving antiretroviral therapy (ART) in low and middle income countries (LMICs) have unknown treatment outcomes and are classified as lost to follow-up (LTFU). Physical tracing of patients classified as LTFU is common; however, effects of tracing on outcomes remains unclear. The objective of this systematic review is to compare estimates of LTFU, mortality and retention in LMIC in cohorts of patients with and without physical tracing.

**Methods and Findings:**

We systematically identified studies in LMIC programmatic settings using MEDLINE (2003–2011) and HIV conference abstracts (2009–2011). Studies reporting the proportion LTFU 12-months after ART initiation were included. Tracing activities were determined from manuscripts or by contacting study authors. Studies were classified as “tracing studies” if physical tracing was available for the majority of patients. Summary estimates from the 2 groups of studies (tracing and non-tracing) for LTFU, mortality, stop of ART, transfers out, and retention on ART were determined. 261 papers and 616 abstracts were identified of which 39 studies comprising 54 separate cohorts (n = 187,666) met inclusion criteria. Of those, physical tracing was available for 46% of cohorts. Treatment programs with physical tracing activities had lower estimated LTFU (7.6% vs. 15.1%; p<.001), higher estimated mortality (10.5% vs. 6.6%; p = .006), higher retention on ART (80.0 vs. 75.8%; p = .04) and higher retention at the original site (80.0% vs. 72.9%; p = .02).

**Conclusions:**

Knowledge of patient tracing is critical when interpreting program outcomes of LTFU, mortality and retention. The reduction of the proportion LTFU in tracing studies was only partially explained by re-classification of unknown outcomes. These data suggest that tracing may lead to increased re-engagement of patients in care, rather than just improved classification of unknown outcomes.

## Introduction

In response to the global HIV epidemic, a public health approach to antiretroviral therapy (ART) has been widely implemented in low- and middle-income countries (LMICs). In 2010, 6.6 million adults and children received ART, representing a 22-fold increase from 2001 [1]. The rapid scale-up of ART is an impressive public health achievement that has led to dramatic declines in HIV related morbidity and mortality [1]–[4].

Frequently reported outcomes for populations receiving ART include the number of: patients alive and on ART, deaths, patients transferring care from one facility to another (‘transfer out’), patients stopping ART (either physician directed or patient initiated) but remaining in care, and patients lost to follow-up (LTFU). [5]–[8] LTFU is a generic term referring to patients who initiate ART but who have unknown treatment outcomes. These unknown treatment outcomes may be divided into 3 general categories: unreported deaths, unknown transfer of care to a different facility without documentation, and disengagement from care [9].

Patient tracing is a commonly used method to improve retention in care and reduce unknown outcomes. Typically in LMICs, tracing involves contacting patients by telephone (telephone tracing), physically visiting their place of residence (physical tracing), or a combination of both. Tracing patients has two potential benefits: 1) linking patients who are disengaged from care back into the health care system, and 2) improved classification of unknown outcomes. By minimizing the number of individuals who disengage from care, programs optimize care by maintaining the greatest possible number of patients on ART, thus decreasing mortality [9] and complications of immunodeficiency. Additionally, patients who have disengaged from care are at increased risk of transmitting HIV due to uncontrolled viremia [10] and for the selection of drug resistance by virtue of ART treatment interruptions [11], [12]. Maximizing the number of patients alive and receiving ART and minimizing the number of patients with unknown outcomes should become an increasingly important public health priority [1], [13], [14].

Program managers are frequently required to report estimates of LTFU, mortality, and retention to ministries of health, funders, and international organizations [5]–[8]. Furthermore, clinicians, program managers and researchers routinely report on LTFU, mortality and retention to quantify the extent of this issue in LMICs [15]–[17]. Patient tracing may result in the improved classification of unknown outcomes allowing for more accurate estimates of LTFU, mortality, and retention. However, the extent to which patient tracing impacts estimates of LTFU, mortality and retention in LMIC remains uncertain. To the authors’ knowledge the only review that stratifies any of these outcomes by tracing status was a mortality estimate from the Antiretroviral Therapy in Lower Income Countries (ART-LINC) Collaboration [18]. All other identified reviews[14], [19]–[21] have synthesized data from multiple studies without incorporating the potential for patient tracing activities to affect estimates of LTFU, mortality or retention.

The proportion of individuals LTFU one year after the initiation of ART has been reported as high as 25–50% in LMICs [22]–[26]. Reasons for LTFU are multi-factorial and include both program and patient factors. Reported predictors of LTFU include evidence of poor nutrition, low CD4 count at diagnosis, the number of doctors available to treat patients, the ability to contact the patient by telephone and decreased levels of community support. [27]–[29] Additional factors such as patient refusal to take ART, adverse events or toxicity related to medication or alternative priorities may also lead to disengagement from ART programs. Furthermore, poor data recording and reporting and information systems that do not permit communication between ART clinics may contribute to high levels of reported LTFU. A lack of communication between record keeping systems may be particularly relevant in settings where different systems are used or unique national ART patient identifiers are not available leading to an inability to identify patients who have transferred out or died. Additionally, in many LMICs deaths go unreported to national death registries, if they exist, and ART programs lack a consistent link between death registries and reporting of population level ART outcomes.

The objective of this systematic review is to compare summary estimates of LTFU, mortality and retention in LMIC, in cohorts of patients with and without physical tracing. In settings with tracing, we hypothesized that summary estimates of LTFU would decrease and estimates of mortality and retention would increase.

## Methods

The strategy to identify appropriate studies, abstract data from selected studies and an analytic plan was established in a systematic review protocol.

### Search Strategy

All searches were performed using Ovid MEDLINE. Searches were limited to studies published in English from January 2003 through May 2011. Studies assessing outcomes in children (<13 years old) were excluded. The search strategy started by combining all sets of terms under the following Medical Subject Headings (MeSH) to identify HIV infected participants receiving ART: “HIV” or “HIV Infections” or “Antiretroviral Therapy, Highly Active” or “Anti-Retroviral Agents”. Then to identify studies from LMICs we combined all sets of terms under the following MeSH: “Africa” or “Asia” or “Caribbean region” or “Central America” or “Latin America” or “South America”, in addition to the following terms: “resource limited” or “resource constrained” or “developing countries” or “low income countries” or “low and middle income countries” or “Africa” or “Afrika” or "sub Saharan" or “southern Africa” or “Asia” or “Latin America” or “South America”. Terms specific to Eastern Europe were not included. The next step combined different combinations of “lost (or loss) to follow up”, with the terms: “attrition” or “retention”, and all terms under the MeSH “patient dropouts”. Finally, items obtained from the searches for: HIV infected participants, LMICs and Loss to follow-up were combined. The exact search strategy is available within the systematic review protocol provided as a supporting document to this manuscript.

The online conference abstract databases for the 2009 International AIDS Society Conference on HIV Pathogenesis, Treatment and Prevention, the 2010 International AIDS Conference, and the 2009–2011 Conference on Retroviruses and Opportunistic Infections were searched for the terms “lost (or loss) to follow up” and “retention”. These more recent years were chosen to capture additional data reported in abstract form that may not have been published in peer reviewed journals. Reference lists from recent reviews assessing patient retention in ART programs in LMICs were also searched [14], [19].

### Study Selection

Original research studies or abstracts reporting on outcomes of HIV infected patients receiving ART in LMICs were included. Studies were included if they were specifically designed to report on LTFU or in cases where it was a secondary finding. Study designs were either cross-sectional or cohort and either prospective or retrospective. All studies included in the analyses reported rates of LTFU for cohorts of individuals who had received care for 12 months after ART initiation and any definition of LTFU was accepted. If cohort studies only reported a median duration of follow up, they were included only if the duration of follow up ranged from 9 to 15 months. When more than one study reported on the same cohort of patients, only the publication containing the most detailed information was included.

**Figure 1:**
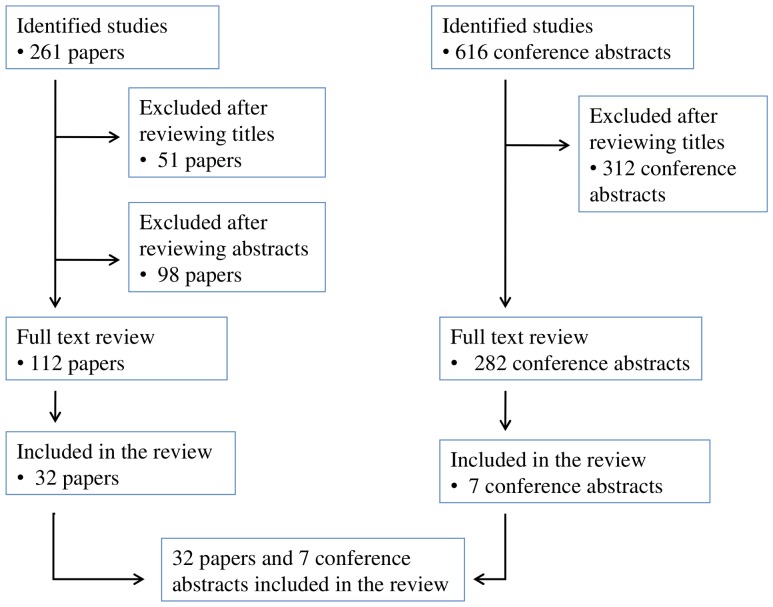
Search strategy and study selection.

Studies in which the majority of patients were children, patients received mono- or dual-therapy, or that were not performed in LMICs were excluded. Additionally, clinical trials were excluded as the focus of this review was to understand LTFU in service delivery settings. Studies were excluded at one of three steps: after review of the title, the abstract, or the manuscript. The search strategy and study selection is summarized in Figure 1.

### Data Abstraction and Management

The following data were abstracted from each study: first author, year of publication, country or countries, healthcare setting (public, private, non-governmental organization), need to pay for ART, dates observed, number of clinics, number of patients receiving ART, baseline demographics (age, gender, CD4 count, clinical stage), ART regimen, ART naive prior to ART initiation, study definition of LTFU and the proportion of patients meeting that definition 12 months after initiation of therapy. If reported, the proportion of subjects who died, transferred care to a different facility, or who stopped ART was abstracted. In addition, details of patient tracing were abstracted, and to minimise reporting bias across selected studies, authors not reporting on patient tracing activities were contacted to establish details about tracing. To provide consistency across all studies the denominator of LTFU estimates included all patients who initiated ART.

### Data Analysis

Proportions of patients classified as LTFU, died, stopped ART and transferred to a different facility were derived from text, tables and graphs (if exact values were available) within studies. Data presented as incidence density (e.g. person years) were converted to cumulative incidence using standard formulae [30]. Patient ‘retention on ART’ was defined as patients alive and receiving ART at the original site plus the group of patients who have ‘transferred out’. This assumes that patients who are known to have transferred their care to another site providing ART are retained in care. The proportion retained on ART was determined for studies that reported at least the proportion LTFU and proportion died using the following formula: Retained on ART = 1–LTFU - died - stopped ART. Additionally, the term ‘retention at the original site’ defines individuals retained on ART and excludes those who have transferred out. Studies also reporting the proportion transferred out were used to estimate the proportion retained at the original site using the formula: Retained at the original site = 1–LTFU–died–stopped ART–transfer out. For the purpose of this review, if transfer out data were not available for a cohort, the estimates of retained on ART and retained at the original site would be the same, an approach consistent with previous reviews focusing on retention in ART treatment programs [14], [19]. Summary estimates for tracing and non-tracing studies are reported as medians if the group of estimates was non-normally distributed or as weighted means if data were normally distributed. Weighting of each proportion derived from included studies was by the inverse of its variance [1 / (p x (1-p) / n), where p is the proportion and n is the sample size]. Tracing was deemed to have occurred if the activity involved physical tracing of the patient with unknown outcome to her or his residence and if this tracing activity was performed for at least one half of the study population. Non-physical tracing studies may have reported no tracing activities or phone tracing only. When choosing a method to differentiate tracing from non-tracing studies we elected to compare physical versus non-physical tracing studies due to the potential for the face-to-face interaction associated with attending a place of residence to increase the chances of re-engagement into care.

Summary estimates from the 2 groups of studies (tracing and non-tracing) were compared by the Student’s t-test if normally distributed, or the Wilcoxon rank sum test if non-normally distributed for each parameters of interest (LTFU, death, stop of ART, transfer to another facility and retention on ART). A Shapiro-Wilk test p value > 0.05 was used to classify estimates from the tracing and non-tracing groups of studies as normally distributed. No assessment of risk of bias was performed for selected studies. Analyses were conducted using Excel and SAS v9.2 (SAS Institute, Cary, NC).

## Results

A total of 261 papers and 616 conference abstracts were identified by the search strategy and of these 39 studies, 32 papers and 7 conference abstracts, met inclusion criteria[4], [16], [17], [22]–[24], [27], [31]–[62] leading to 54 separate cohorts (47 cohorts in 32 papers, and 7 cohorts in 7 abstracts) available for analysis. In 3 papers, data were available for more than one cohort [17], [56], [57] with 2 of these papers reporting on cohorts with and without physical tracing, [17], [57] while the third paper provided data for 2 cohorts that both performed physical tracing [56]. The 39 included studies reported on 17 countries from sub-Saharan Africa, four multi-country studies from sub-Saharan Africa, three countries from Asia and one from Latin America. Published studies contained information to establish tracing status for 18 of the 54 cohorts included in this review. For the remaining 36 cohorts, tracing status was established by contact with study authors. Table 1 presents data on cohorts with physical tracing and Table 2 on cohorts without physical tracing.

**Table 1:** Cohorts with physical tracing.*.

Study (Year)	Country (Cohort or data source)	Type of care	Free ART	Dates observed	Sites (n)	Start ART (n)	Baseline features (Median age, % male, median CD4, WHO clinicalstage)	ART regimens&	Time since ART start	Study definition LTFU	LTFU (%)	Died (%)	ART stop (%)	TF out (%)
May et al [56] (2010)	Cote d’Ivoire (Abidjan - CEPREF)	NGO	Yes	Initiated Jan 04–Mar 07	Many	2117	35, 26%, 129, 82% advanced	NR	Mean 11 months	Not attend clinic for > 6 months	11.5	8.7	NR	NR
May et al [56] (2010)	Malawi (Lilongwe)	Public	Yes	Initiated Jan 04–Mar 07	1	3028	36, 41%, 127, 96% advanced	NR	Mean 10 months	Not attend clinic for > 6 months	12.4	8.9	NR	14
Thai et al [46] (2009)	Cambodia (Phnom Penh)	Private (non profit)	Yes	Mar 03–Dec 07	1	1667	35, 49.4%, 61, Stage III 39% Stage IV 46%	100% NNRTI	12 months	Not attended clinic for 6 consecutive months	3.9	7.6	NR	NR
Bedelu et al [57] (2007)	South Africa (Lusikisiki)	Public, NGO	Yes	Initiated Jan–Jun 05. F/U to Jul 06	12	595	NR	NR	Median 12 months	NR	2.2	16.8	NR	NR
Etard et al [40] (2006)	Senegal	Public	Partial	Initiated Aug 98–Apr 02. F/U to Sep 05	≥ 3	404	37, 45%, 128, CDC Stage B 39%, CDC Stage C 55%	42% PI 95% ART naïve	12 months	6 months with no contact, or 6 months with no ART if contacted	1.7	11.6	NR	NR
Ferradini et al [41] (2006)	Malawi (Chiradzulu)	Public, NGO	Yes	01–April 04	1	1308	35, 36%, 112, Stage III 55% Stage IV 27%	98% NNRTI 97% ART naïve	12 months	Not attended clinic > 2 months after last scheduled visit	5	19	NR	NR
Marston et al [43] (2007)	Kenya (Kibera)	Public, NGO	Yes	Feb 03–Feb 05	1	283	Mean 36, 30%, 157, Stage III 23% Stage IV 48%	99% NNRTI 1% PI	12 months	No clinic visit in > 3 months	13.0	7.0	NR	NR
Coetzee et al [4] (2004)	Sth Africa (Khayelitsha)	Public, NGO	Yes	Initiated May 01–Dec 02, Censored July 03	3	287	31, 30%, 43, Stage III/IV 100%	99% NNRTI	Median 14 months	Not attended services (clinic or other services) for ≥ 3 months after last scheduled appointment	0.3	13.2	3.1	1.0
Palombi et al [27] (2009)	Mozambique, Malawi. Guinea-Conakry	Public, NGO	Yes	Initiated Feb 02–Jan 06. F/U to Jun 07	5	3749	34, 38%, 192, Stage III/ IV 37%	97% NNRTI 3% 3NRTI	Median 15 months	Not attending clinic for > 3 months	2.8	10.5	NR	NR
DeSilva et al [39] (2009)	Nigeria (Jos)	NGO	Yes	Initiated Dec 04–Apr 06. F/U to Dec 06	1	1552	34, 29%, 112, NR	99% NNRTI 1% PI	Mean 15 months	No clinic records for > 3 months	8.8	6.7	NR	NR
Barth et al [31] (2008)	Sth Africa (Ndlovu)	NGO	Yes	Initiated Sept 03–Apr 06	1	609	35, 29%, 67, Stage III 62% Stage IV 17%	100% NNRTI	12 months	NR	15.0	19.0	NR	NR
Moore et al [44] (2010)	Malawi (Blantyre)	Public	Yes	Initiated 05	1	300	Mean 36, 39%, Mean 157, Stage IV 29%	100% NNRTI	12 months	Failure to attend clinic ≥ 4 weeks after last scheduled appointment	2.7	14.3	5.3	5.3
Mutevedzi et al [45] (2010)	Sth Africa(Kwa-Zulu Natal)	Public	Yes	Initiated Oct 04–Sept 07	16	3010	34–37, 22%, 91–128,NR	100% NNRTI	12 months	No clinic visit for ≥ 90 days	3.7	10.9	NR	1.4
Tassie et al [17] (2010)	Kenya (Busia)	NGO	Yes	Initiated Jan–Dec 05	1	860	NR	NR	12 months	No recorded visit for ≥ 90 days from last visit	8.5	6.4	3.0	NR
Tassie et al [17] (2010)	Kenya (Hornabay)	NGO	Yes	Initiated Jan–Dec 05	1	954	NR	NR	12 months	No recorded visit for ≥ 90 days from last visit	10.9	10.6	1.6	NR
Tassie et al [17] (2010)	Kenya (Kibera)	NGO	Yes	Initiated Jan–Dec 05	1	435	NR	NR	12 months	No recorded visit for ≥ 90 days from last visit	11.3	5.3	5.7	NR
Tassie et al [17] (2010)	Kenya (Mathare)	NGO	Yes	Initiated Jan–Dec 05	1	549	NR	NR	12 months	No recorded visit for ≥ 90 days from last visit	13.5	4.2	5.8	NR
Tassie et al [17] (2010)	Malawi (Thyolo)	NGO	Yes	Initiated Jan–Dec 05	1	1359	NR	NR	12 months	No recorded visit for ≥ 90 days from last visit	8.9	12.7	2.4	NR
Tassie et al [17] (2010)	Nigeria (Lagos)	NGO	Yes	Initiated Jan–Dec 05	1	713	NR	NR	12 months	No recorded visit for ≥ 90 days from last visit	9.7	5.7	1.7	NR
Tassie et al [17] (2010)	Zambia (Kapiri Kawama)	NGO	Yes	Initiated Jan–Dec 05	1	559	NR	NR	12 months	No recorded visit for ≥ 90 days from last visit	3.4	12.0	0.9	NR
Tassie et al [17] (2010)	Zimbabwe (Bulawayo)	NGO	Yes	Initiated Jan–Dec 05	1	222	NR	NR	12 months	No recorded visit for ≥ 90 days from last visit	13.5	11.5	0.5	NR
Tassie et al [17] (2010)	Zimbabwe (Connaught)	Public	Yes	Initiated Jan–Dec 05	1	378	NR	NR	12 months	No recorded visit for ≥ 90 days from last visit	4.0	6.3	1.6	NR
Culbert et al [38] (2007)	Congo (Bukavu)	NGO	Yes	Initiated May 02–Jan 06	1	494	37, 34%, 123, Stage III 49% Stage IV 34%	100% NNRTI	12 months	NR	5.4	7.9	NR	NR
Johannessen et al [60] (2008)	Tanzania (Manyara)	NGO	Yes	Initiated Oct 03–Nov 06	1	320	35, 30%, NR, Stage III 31% Stage IV 66%	100% NNRTI	Mean 11 months	Missed appointments for ≥ 3 months	9.7	29.7	2.2	10.9
Chi et al [35] (2009)	Zambia (Lusaka)	Public	Yes	Initiated Apr 04–Sept 07	18	37039	35, 39%, 110–132, Stage III 59% Stage IV 10%	100% NNRTI	12 months	NR	13.8	9.9	3.1	NR

* Defined as physical tracing to the patients place of residence and was available to at least half the study population.

& All ART naïve at baseline unless stated **Notes:** ART, antiretroviral therapy; WHO, World Health Organization; LTFU, lost to follow up; TF, transfer; NR, not reported; F/U, follow up; NGO, non-governmental organization; NNRTI. Non-nucleoside reverse transcriptase inhibitors; PI, protease inhibitors; NRTI, nucleoside reverse transcriptase inhibitors;

**Table 2:** Cohorts without physical tracing.*.

Study (Year)	Country (Cohort or data source)	Type of care	Free ART	Dates observed	Sites (n)	Start ART (n)	Baseline features (Median age, % male, median CD4, WHO clinicalstage)	ART regimens^&^	Time since ART start	Study definition LTFU	LTFU (%)	Died (%)	ART stop (%)	TF out (%)
Geng et al [16] (2010)	Uganda (Mbarara)	Public	Yes	Initiated Jan 04–Sept 07	1	3628	35,39%, 95, NR	NR	12 months	Not attended clinic for > 6 months	16	1.7	NR	NR
Bisson et al [33] (2008)	Botswana (Gaborone - IDCC)	Public	Yes	Initiated Feb 03–August 03	1	410	37, 40%, 81, NR	97% NNRTI 12% prior ARVs	Median 10 months	Last contact with clinic or pharmacy > 30 days after last scheduled visit	16.6	7.1	NR	NR
Bedelu et al [57] (2007)	South Africa (Lusikisiki)	Public, NGO	Yes	Initiated Jan 05–Jun 05. F/U to Jul 06	1	430	NR	NR	Median 12 months	NR	19.3	13.5	NR	4.0
Wools-Kaloustian et al [24] (2006)	Kenya (Western)	Public	Some paid	Nov 01–Feb 05	8	2059	37, 40%, 86, Stage III 38% Stage IV 17%	95% NNRTI	Median 9 months	Not attended clinic > 3 months	24.5	5.4	NR	NR
Wester et al [48] (2005)	Botswana (Gaborone - IDCC)	Public	Yes	Initiated Apr01–Jan 02. F/U to Nov 03	1	153	36, 41%, 96, Stage III 30% Stage IV 47%	100% NNRTI	12 months	Miss 2 consecutive visits and then not contactable on 2 phone attempts	8.4	15.3	NR	5.2
Charalambous et al [34] (2007)	Sth. Africa	Private (Work)	Yes	Oct 02–Dec 05	69	2262	41, 95%, 158, Stage III 45% Stage IV 27%	“NNRTI”	12 months	"Stopped treatment" = patient request, LTFU or for ART non-adherence	8.3	4.2	See LTFU	5.5
Bisson et al [32] (2006)	Botswana (Gaborone)	Private	Yes	Initiated Dec 99–Jan 04	1	346	37, 42%, 80–113, NR	NR	12 months	No viralload tests after ART start, then not contactable by phone and not picking up ART	12.4	5.2	NR	12.1
Laurent et al [23] (2005)	Cameroon (Douala)	Public/Private	Yes	Oct 00–Dec 03	19	788	39, 48%, 123, CDC stage B 57% CDC stage C 33%	NR 86% ART naïve	Median 13 months	Did not attend in 3 months prior to chart review	25.1	6.6	NR	NR
Karcher et al [42] (2007)	Kenya (Migori)	Public	Yes	Apr 04–Sept 05	1	124	31, 29%, 189, CDC Stage C 46%	“NNRTI”	Median 9 months	Not attended within 4 months after last scheduled appointment	15.3	12.1	NR	NR
Hawkins et al [22] (2007)	Kenya (Nairobi –Saint Mary’s)	NGO	Yes	Initiated Sep 04–Aug 06	1	1286	36, 40.9%, 121	99% NNRTI	Median 12 months	Missed clinic visits and failure to collect ART refills for ≥ 3 months	34.8	1.1	NR	4.9
Chung et al [36] (2010)	Kenya (Nairobi - Coptic Center)	NGO	Yes	Initiated Mar 06–Dec 07	1	1231	NR	NR	12 months	Not clinic visit > 30 days after last scheduled pick-up or no clinic visit in 120 days if no pharmacy data	10.0	NR	NR	NR
Toure et al [47] (2008)	Cote d’Ivoire (Abidjan- not CEPREF])	Public/Private	Partial	Initiated May 04–Feb 07	18	8094#	36, 30%, 123, Stage III 69% Stage IV 12%	94% NNRTI	12 month	Last contact with care center > 3 months and not known to be dead or TF out	18	NR	NR	NR
Collini et al [37] (2009)	Ghana (Kumasi)	Public	Partial	Jan 04–Jan 07	1	237	Mean 40, 41%, Mean 120, Stage III/IV 78%	“NNRTI”	12 months	NR	20.3	NR	NR	NR
Assefa et al [61] (2010)	Ethiopia	Public	Yes	Initiated Sept 03–Oct 07	353	60476	NR	100% NNRTI	12 months	Not on ART and not known to have died at 12 months	18.4	8.6	NR	NR
Hong et al [58] (2010)	Namibia	Public	Yes	Initiated after Jan 07	9	1620	NR	100% NNRTI	12 months	Not returned to pharmacy or clinic < 90 days after ART run-out date and have not TF out, stopped, died	17.5	NR	NR	NR
Sharma et al [62] (2010)	India (Delhi - AIIMS)	Public	Yes	Initiated May 05–Oct 06	1	631	Mean 36, 80%, 110, Stage III 51% Stage IV 31%	100% NNRTI	12 months	NR	18.5	13.0	NR	NR
Tassie et al [17] (2010)	Cambodia (Kampong Cham)	NGO	Yes	Initiated Jan–Dec 05	1	606	NR	NR	12 months	No recorded visit for ≥ 90 days from last visit	4.4	9.6	4.8	NR
Tassie et al [17] (2010)	Cambodia (Phnom Penh)	NGO	Yes	Initiated Jan–Dec 05	1	610	NR	NR	12 months	No recorded visit for ≥ 90 days from last visit	0.8	3.3	8.5	NR
Tassie et al [17] (2010)	Uganda (Arua)	NGO	Yes	Initiated Jan–Dec 05	1	1137	NR	NR	12 months	No recorded visit for ≥ 90 days from last visit	13.4	3.9	2.9	NR
Tassie et al [17] (2010)	India (YRG)	Public/Private	Yes	Initiated Jan–Dec 05	1	767	NR	NR	12 months	No recorded visit for ≥ 90 days from last visit	31.9	3.6	6.0	NR
Tassie et al [17] (2010)	Kenya (AMPATH)	Public	Yes	Initiated Jan–Dec 05	1	4111	NR	NR	12 months	No recorded visit for ≥ 90 days from last visit	15.0	6.7	3.8	NR
O’Brien et al [59] (2009)	Congo (Pool)	NGO	Yes	During 07	2	236	37, 31%, 104, Stage III 53%Stage IV 44%	“NNRTI and PI”	Mean 9 months	NR	8.5	12.3	NR	NR
Chinh et al [52] CROI (2010)	Vietnam (Ho Chi Minh City)	Public	Yes	Initiated Sep 05–Dec 07	1	889	30, 77%, 143, Stage III/IV 51%	NR 76% prior ARV	Median 10 months	NR	4.0	5.0	NR	1.2
Cortes et al [51] CROI (2010)	Chile (Chilean AIDS cohort)	Public	Yes	Oct 01 –Sept 08	29	3045	37, 85%, NR, NR	63% NNRTI 15% PI	12 months	NR	2.3	7.1	NR	NR
Auld et al [55] IAS (2010)	Mozambique (National Sample)	Mixed	NR	04–07	30	2596	34, 38%, 153, NR	88% NRTI or NNRTI	1.3 years	NR	22.8	4.3	NR	NR
Ehmer et al [50] IAS (2009)	Africa+ (Solidarmed)	Mixed	Yes	05–08 initiation	8	4362	38, 35%, 121, Stage III/IV 73%	NR	12 months	NR	13.9	10.3	NR	NR
Somi et al [49] CROI (2011)	Tanzania (National Sample)	Public	Yes	Initiated Oct 04–Aug 07	43	2,781	37, 32%, 114, Stage III/IV 77%	NR	12 months	NR	20.0	5.0	5	NR
Bertagnolio et al [53] CROI (2011)	Africa (Multiple countries)	Public	NR	02–10	6	829	NR	NR	12 months	Not returned to pharmacy or clinic < 90 days after ART run-out date and have not TF out, stopped, died	12.9	9.2	0.8	14.5
Balestre et al [54] CROI 2011	IeDEA-West Africa^	NR	NR	93% of patients after 04	NR	19,131	40, NR, 159, 85% advanced stage	87% NNRTI 85% ART naïve	12 months	NR	32.8	4.3	NR	NR

* Defined as physical tracing to the patient’s place of residence and was available to at least half the study population. Note that 7 cohorts [17], [32], [36], [48], [52], [62] reported phone only tracing was available to a proportion of the study population, and 4 cohorts [34], [47], [54], [58] reported physical tracing for a minority of the study population ^&^ All ART naïve at baseline unless stated.

# Sample size determined via contact with study authors.

^ Benin, Cote d’Ivoire, Gambia, Mali, Nigeria, Senegal.

+ Lesotho, Mozambique, Tanzania, Zimbabwe **Notes:** ART, antiretroviral therapy; WHO, World Health Organization; LTFU, lost to follow up; TF, transfer; NR, not reported; F/U, follow up; NGO, non-governmental organization; NNRTI. Non-nucleoside reverse transcriptase inhibitors; PI, protease inhibitors; NRTI, nucleoside reverse transcriptase inhibitors.

**Figure 2:**
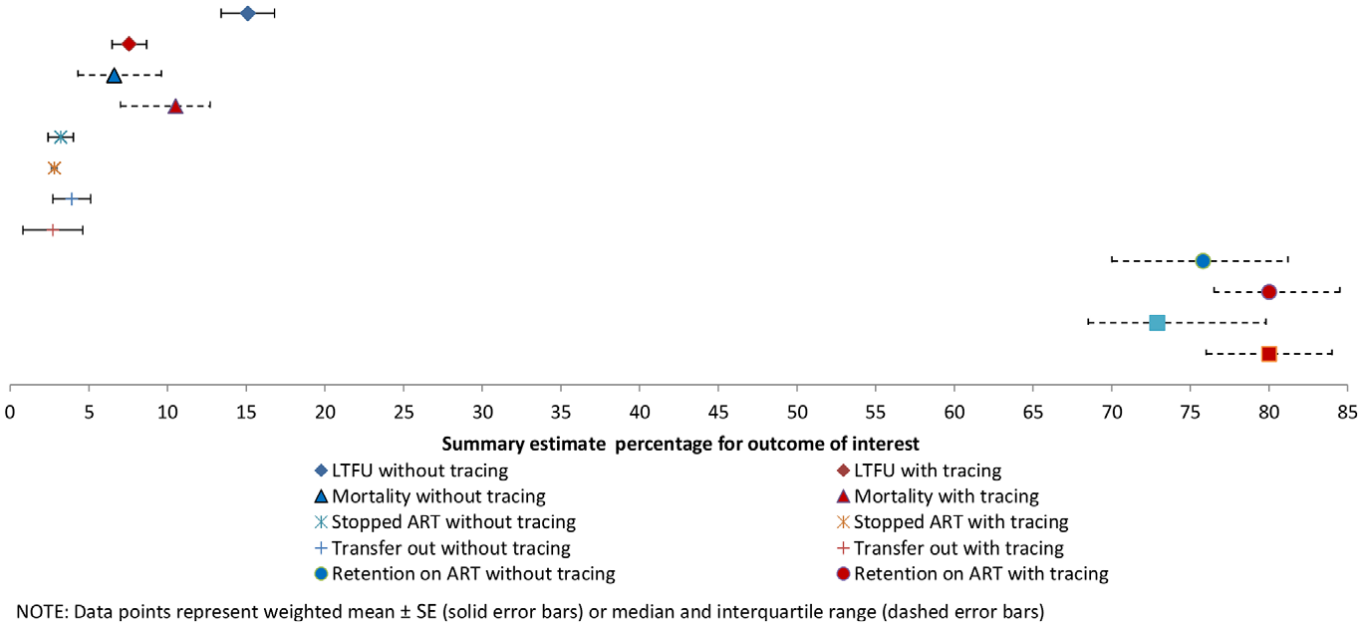
Plot of summary estimates with and without physical tracing.

In the 25 cohorts with physical tracing, the weighted mean LTFU was 7.6 % (SE ± 1.1%, range 0.3–15.0%). In the 29 cohorts without physical tracing, the weighted mean LTFU was 15.1% (SE ± 1.7%, range 0.8–34.8%) (Table 3 and Figure 2). The observed difference in summary estimates was statistically significant (p<0.001). Definitions of LTFU were different across different studies but 52% of cohorts (28/54) classified patients as LTFU 3–4 months after their last contact with the ART clinic. Five studies required a 6 month period of being lost and seven used a variety of definitions. Estimates of mortality were significantly higher (p = .006) in cohorts where physical tracing occurred; median estimate of 10.5% (IQR 7.0–12.7%, range 4.2–29.7%,) compared to 6.6% (IQR 4.3–9.6%, range 1.1–15.3%) in cohorts without physical tracing. Weighted mean estimates of ART stop were 2.8% (SE ± 0.2%, range 0.5–5.8%) in the 13 cohorts with physical tracing compared to 3.2% (SE ± 0.8%, range 0.8–8.5%) in the seven cohorts without physical tracing (p = 0.5). The weighted mean estimate of transfer out to another facility was 2.7% (± 1.9%, range 1.0–14.0%) in the five cohorts with tracing and 3.9% (SE ± 1.3%, range 1.2–14.5%) in the seven cohorts without tracing (p = 0.6). A median 80.0% of patients were retained on ART in studies reporting physical tracing (IQR 76.5–84.5%, range 58.4–88.5%) versus 75.8% (IQR 70–81.2%, range 58.5–91.0%) in studies without physical tracing. The median of retention in care at the original ART site for cohorts with physical tracing was 80.0% (IQR 76.0–84.0%, range 47.5–88.5%) versus 72.9% (IQR 68.5–79.8%, range 58.5–90.6%) at clinics without physical tracing. Differences in retention were statistically significant, p = .04 for retention on ART and p = .02 for retention at the original site.

**Table 3:** Comparison of summary estimates with and without physical tracing.

Outcome of interest	With tracing				Without tracing				P value#
	Cohorts (n)	Starting ART (n)	Range of estimates (%)	Summary estimate* (%)	Cohorts (n)	Starting ART (n)	Range of estimates (%)	Summary estimate* (%)	
**LTFU**	**25**	**62791**	**0.3**–**15.0**	**7.6 ± 1.1**	**29**	**124875**	**0.8**–**34.8**	**15.1 ± 1.7**	**< 0.001**
**Mortality**	25	62791	4.2–29.7	10.5 (7.0–12.7)	25	113693	1.1–15.3	6.6 (4.3–9.6)	**0.006**
**Stopped ART**	13	43975	0.5–5.8	2.8 ± 0.2	7	10841	0.8–8.5	3.2 ± 0.8	**0.5**
**Transfer out**	5	6945	1.0–14.0	2.7 ± 1.9	7	6195	1.2–14.5	3.9 ± 1.3	**0.6**
**Retention on ART**	25	62791	58.4–88.5	80.0 (76.5–84.5)	25	113693	58.5–91.0	75.8 (70.0–81.2)	**0.04**
**Retention at original site**	25	62791	47.5–88.5	80.0 (76.0–84.0)	25	113693	58.5–90.6	72.9 (68.5–79.8)	**0.02**

* Values represent median (Q1–Q3), or weighted mean ± SE (estimates weighted by the inverse of their variance).

# Comparing summary estimates for the 2 groups of studies (tracing and non–tracing) by Wilcoxon rank-sum test for medians or student’s t test for weighted means **Notes:** LTFU, lost to follow up; ART, antiretroviral therapy.

## Discussion

This review demonstrates lower estimates of LTFU and higher estimates of mortality in LMIC settings where patients receiving ART attend clinics employing physical tracing. The observed differences may be explained by more accurate classification of patients in studies where physical tracing was performed. Specifically, many patients who had previously been classified as LTFU, once traced, were found to have died, thereby contributing to an apparent increased mortality. It remains uncertain by how much the observed decrease in LTFU within physical tracing cohorts was a result of re-engagement of patients back into care versus re-classification of patients with previously unknown outcomes. However, in addition to the significant reduction in LTFU and increase in mortality, we report a significant improvement in retention at the original site. While it is not unexpected that tracing activities would decrease the proportion LTFU and increase mortality estimates due to improved classification of outcomes, the observed improvement in retention at the original site suggests that tracing may have increased the number of patients re-engaged in care. Although the tracing activity would re-classify patients previously thought to be LTFU as transferred out our having died, this reclassification would not alter the estimate of retention at the original site due to its inclusive definition. Therefore, the improvement in retention at the original site is likely not explained by re-classification, but is due to less LTFU, death, or transfers out. Individuals that are re-engaged would have the opportunity to receive the beneficial effects of ART such as improved survival, decreased risk of opportunistic infections, [63] and potentially preventing virological failure and the emergence of HIVDR by limiting treatment interruptions [11], [12]. In addition, the maintenance of an increased proportion of individuals in care and receiving ART is likely to benefit the community by decreasing HIV incidence [64]–[66]. Furthermore, if data on the costs of physical tracing can be obtained, this intervention may potentially be a cost-effective mechanism to re-engage patients into care. Cost-effective analyses of intervention to minimize LTFU and improve survival have been performed but analyses incorporating tracing are not known to the authors at this time. While the qualifications of individuals performing physical tracing is not always reported some included studies did document tracing by peer supporters or people living with HIV without medical qualifications [27], [39], [57] suggesting that physical tracing may prove cost effective in many settings.

The considerable difference in summary estimates between physical and non-physical tracing emphasizes the importance of knowing whether physical tracing is used within an ART program or at a specific ART clinic when interpreting LTFU, mortality or retention data. Estimates of mortality and LTFU are frequently used to assess level of ART program and clinic performance; [5], [6] thus, understanding differences which arise due to physical tracing are important. In addition, the indicator of retention on ART after 12 months of therapy is considered an essential and high impact information when assessing ART program performance [7], [8]. Criticising a program that does not achieve targets for mortality but has functioning tracing programs resulting in few patients with unknown outcomes may not be appropriate. Likewise, reinforcing current practice in settings without tracing and reporting low mortality and higher LTFU rates sends an incorrect message. Furthermore, guidance from the literature in this area is limited as only one previous review was identified that stratified a summary estimate by tracing status. This study by Braitstein et al [18] documented 6.4% mortality with physical tracing and 2.3% without in an LMIC setting whereas we report a mortality of 10.5% with tracing and 6.6% without tracing . Additional reviews report higher 12-month mortality estimates that do not take tracing into account; 14% by Gupta et al [20] and a range of 8–26% mortality by Lawn et al [21]. The reasons for differences in these mortality estimates are unclear, but potentially reflect differences in reporting or improved clinical outcomes. For example, the lower estimates of mortality reported by Braitstein et al [18] are obtained from a smaller number of sites within the ART-LINC collaboration. Additional reviews report estimates of 75–80% retention consistent with findings in this review although tracing status was not documented [14], [19].

This systematic review has some limitations. Settings where physical tracing is available may have increased resources for patients resulting in improved outcomes. Summarizing the effect of tracing from randomised trials containing tracing interventions may provide a more accurate assessment of the impact of tracing on LTFU, mortality and retention by eliminating potential confounding associated with better resourced sites. The authors are unaware of published randomised trials of this nature but data from this review supports the development of randomised trials to quantify the benefits of different tracing strategies including the cost-effectiveness of these strategies. There is also a potential publication bias for settings more likely to publish on LTFU. For example programs associated with academic institutions may be more likely to prepare manuscripts and these programs may have different outcomes from other non-academic settings less inclined to publish their results. Another limitation of this analysis was variability of definitions of LTFU. The majority of studies used a definition of LTFU consistent with international recommendations, [5] yet it is unclear how our findings would have differed if alternative definitions of LTFU had been used. Furthermore, studies classified as physical tracing studies potentially have differing mechanisms to physically trace patients (e.g. number of attempts) which could have influenced outcomes, although the objective of the review was to compare cohorts with and without physical tracing without focussing on specific subgroups within the physical tracing group of studies. Findings from this review may also be limited if additional data are available from other biomedical databases or relevant grey literature. If additional unidentified studies have different findings from the 54 cohorts identified through Ovid Medline and the international HIV conference databases, summary findings could be different. Finally, data on transfer out was only available in a minority of studies despite looking for this data in all selected studies. Estimates of retention at the original site could have potentially changed if complete transfer out data were available. For example a potential bias could exist if cohorts with physical tracing had decreased transfers out which was not documented. This could lead to increased estimates of retention at the original site not necessarily explained by increased re-engagement in care. This limitation emphasises the importance of understanding the proportion transferred out when accurately interpreting and comparing estimates of retention.

In conclusion, physical tracing leads to a reduction in unknown outcomes and likely improved re-engagement in care. Findings from the observational data in this review highlight a critical need for randomised controlled trials to support the effectiveness of patient tracing to improve re-engagement of patients on ART and assess the cost-effectiveness of tracing interventions. Programs providing ART in LMICs should consider physically tracing patients who have become disengaged from care as an important intervention to improve individual outcomes and programmatic evaluation of HIV infected populations receiving ART.

## Supporting Information

Material S1 **Physical tracing effects systematic review protocol**
(DOC)
