# Supplementary material for: Effects of Physical Tracing on Estimates of Loss to Follow-Up, Mortality and Retention in Low and Middle Income Country Antiretroviral Therapy Programs: A Systematic Review
Source: PLoS One. 2013 Feb 12;8(2):e56047. doi: 10.1371/journal.pone.0056047 (PMC3570556; doi:10.1371/journal.pone.0056047)
Supplement: Material S1 — Physical tracing effects systematic review protocol (DOC) [file pone.0056047.s001.doc]

**Protocol for the systematic review of the effects of tracing on estimates of lost to follow-up, mortality and retention in antiretroviral therapy programs**

**1. Authors**

James H. McMahon

Alfred Hospital, Melbourne, Australia

Tufts University School of Medicine, Boston, USA

Julian H Elliott

Alfred Hospital, Monash University, and Burnet Institute, Melbourne, Australia

Steven Y. Hong

Tufts Medical Center and Tufts University School of Medicine, Boston, USA

Silvia Bertagnolio

World Health Organization, Geneva, Switzerland

Michael R. Jordan

Tufts Medical Center and Tufts University School of Medicine, Boston, USA

**2. Background**

The proportion of individuals lost to follow-up (LTFU) is a commonly reported outcome for patients receiving antiretroviral therapy (ART) in low-middle income countries (LMICs). Individuals LTFU are those with unknown outcomes.

Patient tracing allows patients who are LTFU to be re-classified as died, transferred to another ART site or disengaged from care. As well as reducing unknown outcomes tracing also has the potential to re-engage patients and improve retention in care. Tracing activities can involve contacting patients by telephone or physically visiting their place of residence (physical tracing), or a combination of both. Through the minimization of disengagement from care, ART programs have the ability to maintain the greatest possible number of patients on ART. Maximizing engagement has many potential benefits which include: reducing mortality, avoiding the complications of immunosuppression, minimizing the risk of transmitting HIV and the selection of drug resistance by reducing ART treatment interruptions

The extent to which patient tracing impacts estimates of LTFU, mortality and retention in LMIC is unclear. Prior literature reviews have synthesized data from multiple studies without incorporating the potential for patient tracing activities to affect estimates of LTFU, mortality and retention. The Antiretroviral Therapy in Lower Income Countries (ART-LINC) Collaboration did incorporate how tracing effects mortality estimates.

**3. Objective and Hypothesis**

The objective is to compare summary estimates of LTFU, mortality and retention in LMIC, in cohorts of patients with and without physical tracing. The central hypothesis is that summary estimates of LTFU would decrease and estimates of mortality and retention would increase in settings with tracing.

**4. Methods**

Criteria used to consider studies for inclusion:

- May include cross-sectional and cohort studies and may be prospective or retrospective
  - Clinical trials will not be included. Studies will be included if they report on ART programs providing service delivery (i.e. non-research settings)
- Studies included if they are specifically designed to report on LTFU or where it was a secondary finding
- Studies conducted in LMICs (Africa, Asia, Latin America)
  - Terms include “resource limited” or “resource constrained” or “ developing countr*” or “low income countr*” or “low and middle income countr*”
  - Also include africa* or afrika* or "sub sahara*" or southern africa* or asia* or latin america* or south america*
- Participants receiving combination (≥ 3 agents) ART
  - “HIV” or “AIDS” or “human immunodeficiency virus” or “acquired immunodeficiency syndrome” AND “antiretroviral therapy” or “antiretroviral*” or “HAART” or “ART”
- Published in English
- Studies published after January 1, 2003
- Exclude studies of children (< 13 years old)
- Studies reporting on patient lost to follow up after 12 months of ART
  - Duration of follow up has to be defined. If reports median duration only this has to be between 9 and 15 months
  - Include the terms “attrition” or “lost to follow-up” or “loss to follow-up” or "late patients" or "dropout" or "drop-out" or “retention”
- See Appendix 1 for Medline search

Search methods for identification of studies

- Electronic searches.
  - Ovid MEDLINE (through PubMed)
  - Online conference abstract databases for the International AIDS Society (IAS) Conference on HIV Pathogenesis, Treatment and Prevention; the International AIDS Conference and the Conference on Retroviruses and Opportunistic Infections (CROI) from 2009 to 2011
  - CROI
    - Review titles of all oral and poster sessions and review all abstracts in sessions that based on session title (e.g. relevance to LMICs) could contain abstracts of interest (once abstract number found, then review with search function)
  - IAS
    - Search abstracts in “program at a glance” using key terms as defined above (covers abstracts presented in oral sessions)
- Reference lists from recent reviews assessing patient retention in ART programs in LMICs were also searched

Data collection and analysis

- Selection of studies
  - Retrieved titles and abstracts will be assessed for full-text review using the criteria described above

Data extraction and management

- The following data will be abstracted to a Microsoft Excel spreadsheet:
  - Author, Journal, Year
  - Setting: countries, health facility level, type (public, private) and number
  - Number of study participants
  - Design
  - Characteristics of study population (age, gender, median baseline CD4)
  - ART regimens and proportion of population non-ART naïve
  - Study definition of LTFU
  - Proportion meeting study definition of LTFU
  - If available: proportion died, proportion transferred care to another ART site, proportion stopped ART (either physician directed or patient initiated)
  - Details of patient tracing activities
- When more than one study reported on the same cohort of patients, include the publication containing the most detailed information
- Authors of studies not reporting on patient tracing activities to be contacted to establish tracing details
  - A maximum of 3 attempts by email to the corresponding and/or senior author

**5. Statistical analysis and data synthesis**

- Proportions LTFU, died, stopped ART and transferred out abstracted from text, tables and graphs (if exact values were available)
- The proportion retained on ART is determined for studies that reported at least the proportion LTFU and proportion died using the following formula: Retained on ART = 1 – LTFU - died - stopped ART.
- If studies also report the proportion transferred out the proportion retained at the original site will be established using the formula: Retained at the original site = 1 – LTFU – died – stopped ART – transfer out.
  - Therefore, if transfer out data were not available for a cohort, the estimates of retained on ART and retained at the original site would be the same. This is consistent with previous similar reviews of retention in ART programs .
- Summary estimates of LTFU, mortality, retention on ART and retention at the original site for tracing and non-tracing studies will be established and compared by appropriate statistical tests
- Weighting of each proportion that will contribute to summary estimates will be by the inverse of its variance [1 / (p x (1-p) / n), where p is the proportion and n is the sample size].
- Tracing will have occurred if the activity involved physical tracing of the patient to his residence and if this tracing activity was available for at least one half of the study population.
  - Therefore non-physical tracing studies may have no tracing or phone tracing only.

**References:**

1. Fox MP, Rosen S (2010) Patient retention in antiretroviral therapy programs up to three years on treatment in sub-Saharan Africa, 2007-2009: systematic review. Tropical Medicine & International Health 15 Suppl 1: 1-15.

2. Rosen S, Fox MP, Gill CJ (2007) Patient retention in antiretroviral therapy programs in sub-Saharan Africa: a systematic review. PLoS Medicine / Public Library of Science 4: e298.

3. Gupta A, Nadkarni G, Yang WT, Chandrasekhar A, Gupte N, et al. (2011) Early mortality in adults initiating antiretroviral therapy (ART) in low- and middle-income countries (LMIC): a systematic review and meta-analysis. PLoS One 6: e28691.

4. Lawn SD, Harries AD, Anglaret X, Myer L, Wood R (2008) Early mortality among adults accessing antiretroviral treatment programmes in sub-Saharan Africa. AIDS 22: 1897-1908.

5. Braitstein P, Brinkhof MW, Dabis F, Schechter M, Boulle A, et al. (2006) Mortality of HIV-1-infected patients in the first year of antiretroviral therapy: comparison between low-income and high-income countries. Lancet 367: 817-824.

**APPENDIX 1 - Search Strategy**

--------------------------------------------------------------------------------

1 exp Antiretroviral Therapy, Highly Active/ (13213)

2 exp HIV Infections/ (198617)

3 exp HIV/ (70704)

4 exp Anti-Retroviral Agents/ (51271)

5 1 or 2 or 3 or 4 (237850)

6 (resource limited or resource constrained or developing countr* or low income countr* or "low and middle income countr*").tw. (32450)

7 (africa* or afrika* or "sub sahara*" or southern africa* or asia* or latin america* or south america*).tw. (166542)

8 exp Africa/ (151724)

9 exp Asia/ (394665)

10 caribbean region/ or central america/ or latin america/ or south america/ (15057)

11 6 or 7 or 8 or 9 or 10 (657853)

12 exp African Americans/ (33865)

13 exp Asian Americans/ (4265)

14 12 or 13 (37133)

15 11 not 14 (638182)

16 lost to follow up.tw. (9266)

17 lost to followup.tw. (427)

18 loss to follow up.tw. (1249)

19 loss to followup.tw. (22)

20 retention.tw. (93003)

21 attrition.tw. (4883)

22 exp Patient Dropouts/ (5674)

23 16 or 17 or 18 or 19 or 20 or 21 or 22 (112858)

24 5 and 15 and 23 (478)

25 limit 24 to (english language and yr="2003 -Current") (376)

26 limit 25 to clinical trial, all (80)

27 25 not 26 (296)

28 limit 27 to ("all infant (birth to 23 months)" or "preschool child (2 to 5 years)" or "child (6 to 12 years)") (59)

29 27 not 28 (237)
